# A new relative tumor sizing method in epi-metaphyseal osteosarcoma

**DOI:** 10.1186/s12885-015-1129-9

**Published:** 2015-04-15

**Authors:** Seung Hyun Kim, Kyoo-Ho Shin, Eun Hae Park, Yong Jin Cho, Byoung-Kyu Park, Jin-Suck Suh, Woo-Ick Yang

**Affiliations:** 1 Department of Clinical Science, Orthopaedic Surgery, Yonsei University Graduate School, Seoul, Korea; 2 Department of Orthopaedic Surgery, Andong Hospital, Andong-si, Gyeongbuk Korea; 3 Department of Orthopaedic Surgery, Yonsei University College of Medicine, 50 Yonsei-Ro, Seodaemun-Gu, Seoul, 120-752 Korea; 4 Department of Radiology and Research Institute of Radiological Science, Yonsei University College of Medicine, Seoul, Korea; 5 Department of Pathology, Yonsei University College of Medicine, Seoul, Korea

**Keywords:** Osteosarcoma, Relative tumor sizing, Tumor axial ratio

## Abstract

**Background:**

The goal of this study was to develop a new method for determining tumor size to predict prognosis with high performance in osteosarcoma.

**Methods:**

This study was approved by the institutional review board. We retrospectively reviewed 41 magnetic resonance (MR) images at diagnosis and 57 MR images after neoadjuvant chemotherapy from 59 patients with non-metastatic, high-grade extremity osteosarcoma, who had undergone surgery between October 1994 and October 2009.

**Results:**

A new parameter of tumor axial ratio (TAR) was designed to normalize tumor size by dividing the absolute tumor axial size by the reference bone axial size (RBS) of the affected bone. RBS was defined using anatomical landmarks for each type of bone. Absolute tumor length (ATL), absolute tumor volume (ATV), and relative tumor volume (RTV) were comparatively analyzed. TAR was only significantly decreased after chemotherapy in the survival (*P* = 0.009) and metastasis-free (*P* = 0.018) group in the paired *t*-test. With the Kaplan–Meier method, significant differences in overall survival (log rank *P* = 0.004) and disease-free survival (Log Rank *P* = 0.009) were noted between decreased TAR after chemotherapy and increased TAR. After Cox regression analysis, TAR showed an odds ratios of 5.931 for survival (95% Confidence Interval [CI], 1.153–30.513) and 14.144 for metastasis (95% CI, 2.826–70.784), whereas ATL, ATV, and RTV showed no associations with these clinical variables. The AUC value of TAR was 0.713 (95% CI, 0.548 to 0.878) for survival and 0.759 (95% CI, 0.608 to 0.909) for metastasis.

**Conclusions:**

TAR is a novel sizing method with potential as a prognostic tool in osteosarcoma.

## Background

The size of a primary tumor is the most fundamental parameter for staging solid tumors. In bone sarcomas, the American Joint Committee on Cancer (AJCC) 7th edition (2010) has established 8 cm in greatest dimension as the cutoff for subclassification of stage A or B [1]. Besides AJCC staging, several studies have suggested various tumor sizing methods and cutoff points with prognostic powers in osteosarcoma [2-8].

Controversy over methods used to measure sizes of bone sarcomas has arisen from two major problems. First, there is concern over the performance of imaging devices. Prior to magnetic resonance imaging (MRI), the only two-dimensional (2D) sizing methods on roentgenological images did not show significant results [9]. However, significant relationships between tumor burden (tumor volume) and prognosis have been reported for tumor volume based on MRI [10]. Second, size heterogeneity of the affected organ is another major concern that is unique to bone sarcomas compared with other cancers. The sizes of different types of bones vary (e.g. humerus, femur, tibia, and fibula). Furthermore, the same type of bone varies in size depending on sex and age. Size heterogeneity is a serious concern because bone sarcomas mainly occur in children and teenagers. For this reason, the need for a better method to determine relative tumor size is important.

The mostly widely used method for adjusting tumor size is to normalize tumor burden according to individual patient size (body surface area [BSA]), as described by Bieling et al. [3]. This method, which uses an ellipsoid formula to calculate tumor volume, was initially applied on plain X-ray images; however modified applications on MRI have also been reported [5,8,11]. In those studies, there were some inherent limitations that are important to consider. First, adjustment by BSA dose not discriminate bias from the different types of bones involved, although it can rule out bias from heterogeneity in individual patient size. Second, tumor volume based on ellipsoid formulas assumes that the shape of tumors is ellipsoidal, but this may not always be true. Advances in MRI have led to more correct measurement of tumor volume. Indeed, reports based on three-dimensional (3D) volumetry of tumors have shown prognostication of tumor burden [4,6]. However, calculating 3D volumetry on MRI is not clinically practical.

The purpose of this study was to develop a new method for determining tumor size with high performance for predicting prognosis. We developed a novel method for determining relative tumor size that focused on the relative axial length of a tumor.

## Methods

### Patients

We retrospectively reviewed 41 MR images at diagnosis and 57 after neoadjuvant chemotherapy from 59 patients with nonmetastatic, high-grade extremity osteosarcoma, who had undergone surgery between October 1994 and October 2009 and analyzed them together with other clinical data. Mean follow-up period was 114.7 months (range, 4.8–240 months). This study was done under a protocol approved by Severance Hospital Institutional Review Boardl. Both MRI at diagnosis and after neoadjuvant chemotherapy were available for 39 patients. MRI after neoadjuvant chemotherapy but not MRI at diagnosis was available for 18 patients. MRI at diagnosis but not MRI after neoadjuvant chemotherapy was available for two patients. The clinical characteristics of 59 patients are listed in Table 1. Sixty-five patients (92.9%) had received neoadjuvant chemotherapy. Patients were treated as follows: 22 received combination of intraarterial cisplatin and doxorubicin, while 37 received combination of intraarterial cisplatin, doxorubicin, and ifosfamide. Outcomes of neoadjuvant chemotherapy were not significantly different between doublet and triplet regimens in our cohorts [12].

**Table 1:** **Clinical characteristics**

Variable	n (%)	
5-year survival	Yes	43 (72.9)
	No	16 (27.1)
Metastasis rate	Free	41 (69.5)
	Positive	18 (30.5)
Age, mean (range)	17.8 (3–59)	
Sex	Male	32 (54.2)
	Female	27 (45.8)
AJCC stage	IIA	22 (37.3)
	IIB	37 (62.7)
Site	Distal femur	32 (54.2)
	Proximal tibia	16 (27.1)
	Proximal humerus	8 (13.6)
	Proximal femur	3 (5.1)
Histology	Osteoblastic	37 (62.7)
	Chondroblastic	7 (11.9)
	Fibroblastic	4 (6.8)
	Mixed	8 (13.6)
	NA	3 (5.1)
Huvos grade	I and II	16 (27.1)
	III and IV	43 (72.9)
Resection margin	R0	58 (98.3)
	R1	1 (1.7)
ALP	Elevation	33 (55.9)
	Normal	26 (44.1)

*Abbreviations*: *AJCC* American Joint Committee on Cancer, *ALP* Alkaline phosphatase.

### Measurement of tumor size and novel parameter for tumor sizing

All parameters for tumor sizing were measured on MR images. Parameters for tumor sizing were defined as follows: absolute tumor length (ATL), the greatest longitudinal tumor length on either coronal or sagittal images; absolute tumor axial size (ATA), the greatest horizontal tumor length in any direction on axial images. Reference bone axial size (RBS) was defined using anatomical landmarks for each type of bone around the epi-metaphyseal area (Table 2). Measurement of all parameters was done with the Centricity Radiology RA1000 program (General Electrics Healthcare, United Kingdom). All parameters mentioned above were measured independently by three orthopaedic surgeons. Absolute tumor volume (ATV) was measured by one radiologist using 3D region of interest (ROI) magnetic resonance volumetry [13,14].

**Table 2:** **Definitions of the RBS for each bone**

	Definition of RBS
Distal femur	Longest length of the transepicondylar line^†^
Proximal tibia	Longest length of the tibia plateau^†^
Proximal humerus	Longest length of the anatomical neck of the humoral head^†^
Proximal femur	Longest length of the epiphyseal plate or vestigium of the femoral head epiphyseal plate^†^

^†^length measured in coronal plane of MRI.

**Figure 1:**
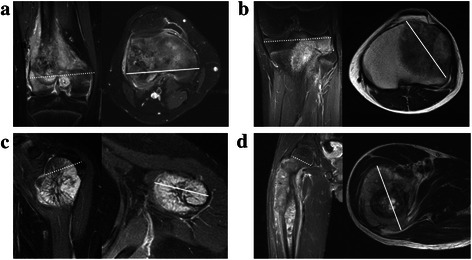
Illustrations for applications of new relative tumor sizing method. Dotted lines indicated RBS of each bone and solid lines indicate ATA of tumors. RBS is defined in Table 2. ATA was defined as the greatest horizontal tumor length in any direction on axial images. TAR was calculated by dividing ATA by RBS. **(a)** RBS of distal femur that was 89.5 mm, ATA of tumor that was 84.2 mm, and TAR of this case was 0.94 (84.2/89.5) **(b)** RBS of proximal tibia that was 77.2 mm, ATA of tumor that was 60.2 mm, and TAR of this case was 0.78 **(c)** RBS of proximal humerus that was 42.5 mm, ATA of tumor that was 44.7 mm, and TAR of this case was 1.05 **(d)** RBS of proximal femur that was 42.3 mm, ATA of tumor that was 67.6 mm, and TAR of this case was 1.59.

A new parameter for tumor sizing, tumor axial ratio (TAR), was designed to normalize tumor size by dividing ATA by RBS for each affected bone; thus, TAR represented the ratio of tumor axial size to affected bone axial size. The applications of our method are illustrated in Figure 1. For example, as shown in Figure 1a, distal femur with a RBS of 89.5 mm, and an ATA of 84.2 mm, the TAR would be 0.94 (84.2/89.5). Other applications for proximal tibia (Figure 1b), proximal humerus (Figure 1c), and proximal femur (Figure 1d) are also provided.

### Statistical analysis

The reliability on measuring those parameters among different observers was evaluated using the interclass correlation coefficient. Changes in those parameters after neoadjuvant chemotherapy were analyzed by the paired *t*-test. Overall survival and disease-free survival between the decreased the TAR group after neoadjuvant chemotherapy and increased TAR were compared using the Kaplan–Meier method and log rank test. The correlations between each parameter and prognosis (5-year survival and metastasis) were expressed as the hazard ratio using Cox regression. Prognostication of TAR and ATV was compared by evaluating the area under the receiver operating characteristic curve (AUC).

All statistical analyses were performed using SPSS (version 20.0, SPSS, Inc., Chicago, IL, USA). All *P* values were two-tailed, and *P* < 0.05 was considered statistically significant.

## Results

### New parameters and their reliability

To be accepted as a standard, the reliability of a method should be evaluated. The lengths of ATL, ATA, ASA, and RBS for all MR images enrolled in this study were measured independently by three orthopaedic surgeons and reliability analyses were performed (Table 3). Interclass coefficients (ICCs) for RBS were 0.947 on MR images at diagnosis and 0.965 on MR images after chemotherapy. These were convincing values because RBSs were defined from well-known and widely used anatomical landmarks. ICCs of ATA were 0.725 on MR images at diagnosis and 0.862 on MR images after neoadjuvant chemotherapy. Factors confounding measurement of ATA were peritumoral inflammatory changes and bone edema, which eventually resulted in the lowest ICCs on MR images at diagnosis. The ICC of ATA on MR images after neoadjuvant chemotherapy was increased as bone edema decreased by neoadjuvant chemotherapy. ICCs of TAR were 0.807 on MR images at diagnosis and 0.862 on MR images after neoadjuvant chemotherapy. Although ICCs of TAR were lower than those of ATL, they showed sufficient reliability.

**Table 3:** **Reliability for interobserver variability of various measurements on MRI**

	Mean ± SD				Interclass correlation (95% CI)	*P*
		Observer 1	Observer 2	Observer 3		
At diagnosis	RBS (mm)	53.40	52.67	54.70	0.947	*<0.001*
		±24.38	±24.06	±24.49	(0.905 to 0.972)	
	ATL (mm)	99.27	94.12	92.24	0.909	*<0.001*
		±66.31	±67.84	±65.00	(0.840 to 0.952)	
	ATA (mm)	49.23	55.74	41.24	0.725	*<0.001*
		±20.93	±49.94	±16.19	(0.509 to 0.855)	
	TAR	1.00	0.95	0.82	0.807	*<0.001*
		±0.31	±0.36	±0.23	(0.652 to 0.899)	
After Neoadjuvant Chemotherapy	RBS (mm)	65.75	62.64	65.38	0.965	*<0.001*
		±20.84	±22.16	±21.02	(0.942 to 0.979)	
	ATL (mm)	103.55	100.05	104.83	0.965	*<0.001*
		±61.73	±63.58	±62.41	(0.944 to 0.979)	
	ATA (mm)	50.18	53.24	46.91	0.775	*<0.001*
		±17.64	±26.16	±17.49	(0.640 to 0.865)	
	TAR	0.86	0.99	0.79	0.862	*<0.001*
		±0.38	±0.50	±0.30	(0.775 to 0.919)	

*Abbreviations*: *SD* standard deviation, *CI* confidence interval, *RBS* reference bone axial size, *ATL* absolute tumor length, *ATA* absolute tumor axial size.

### Prognostications of parameters

Changes in parameters for tumor size after neoadjuvant chemotherapy were analyzed by the paired *t*-test (Table 4). Although there were no significant changes in ATL, ATV, and relative tumor volume (RTV) in all groups, ATA and TAR were significantly reduced in the survival group (*P* = 0.011 and *P* = 0.009, respectively) and metastasis-free group (*P* = 0.016 and *P* = 0.018, respectively). ATA and TAR in the mortality group and metastasis-positive group were not affected.

**Table 4:** **Paired t-tests to evaluate responsiveness to neoadjuvant chemotherapy**

		Total		Survival				Metastasis			
				5-year survival		Death		Free		Positive	
		Mean±SD	*P*	Mean±SD	*P*	Mean±SD	*P*	Mean±SD	*P*	Mean±SD	*P*
ATL (mm)	BC	116.46	*0.850*	112.51	*0.679*	126.52	*0.972*	112.01	*0.352*	126.47	*0.781*
		±54.67		±54.07		±57.53		±55.03		±54.85	
	AC	115.63		111.17		127.01		109.21		130.08	
		±56.30		±60.15		±45.61		±60.38		±44.77	
ATV (ml)	BC	174.62	*0.048*	174.27	*0.060*	175.58	*0.485*	174.53	*0.075*	174.83	*0.260*
		±198.28		±220.29		±127.79		±225.03		±121.45	
	AC	119.40		103.58		163.33		104.85		154.30	
		±87.88		±78.88		±101.16		±80.31		±99.56	
RTV (ml/m^2^)	BC	107.86	*0.059*	109.32	*0.074*	103.83	*0.463*	109.49	*0.092*	103.96	*0.241*
		±119.18		±132.50		±76.87		±135.34		±72.47	
	AC	75.47		67.95		96.35		68.88		91.28	
		±51.20		±47.33		±58.53		±48.12		±57.47	
ATA (mm)	BC	60.49	*0.075*	58.06	*0.011*	66.65	*0.380*	57.31	*0.016*	67.62	*0.540*
		±17.11		±18.56		±11.18		±18.47		±11.18	
	AC	56.82		51.78		69.66		51.14		69.62	
		±14.88		±12.96		±11.63		±12.74		±11.09	
TAR	BC	0.89	*0.114*	0.89	*0.009*	0.87	*0.260*	0.85	*0.018*	0.96	*0.670*
		±0.29		±0.32		±0.17		±0.25		±0.35	
	AC	0.84		0.80		0.94		0.77		0.99	
		±0.26		±0.25		±0.25		±0.20		±0.30	

*Abbreviations*: *SD* standard deviation, *BC* before neoadjuvant chemotherapy, *AC* after neoadjuvant chemotherapy, *ATL* absolute tumor length, *ATV* absolute tumor volume, *RTV* relative tumor volume, *ATA* absolute tumor axial size, *TAR* tumor axial ratio.

**Figure 2:**
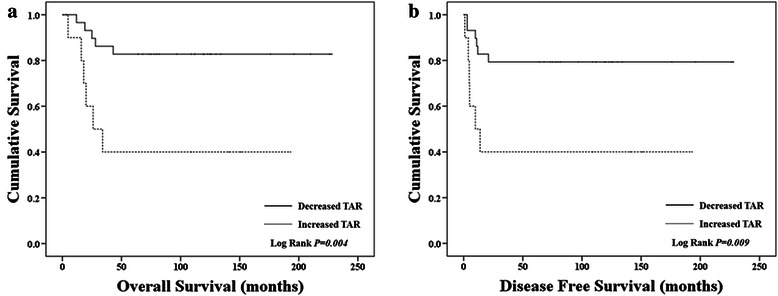
Overall and disease-free survival curves in decreased and increased TAR groups. Significant difference was noted in **(a)** overall survival (Log Rank *P = 0.004*) and **(b)** disease-free survival (Log Rank *P = 0.009*) between the two groups.

Overall survival and disease-free survival in the decreased TAR group after neoadjuvant chemotherapy and increased TAR were compared using the Kaplan–Meier method (Figure 2). Significant differences in overall survival (log rank *P* = 0.004) and disease-free survival (log rank *P* =0.009) were noted.

**Figure 3:**
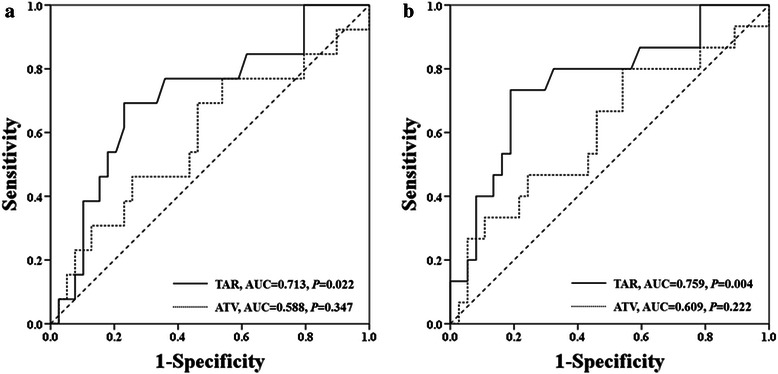
Comparisons on AUC values of TAR and ATV after neoadjuvant chemotherapy for survival and metastasis. ROC curves for **(a)** overall survival and **(b)** disease-free survival.

Prognostication of all parameters was analyzed with respect to 5-year survival and metastasis using Cox regression analysis (Table 5). None of the parameters on MR images at diagnosis showed associations with survival and metastasis. However, parameters for tumor axial extent on MR images after neoadjuvant chemotherapy showed associations with these prognostic variables, whereas the longitudinal extent and volume of tumors did not. ATA showed significant associations with prognosis (*P* < 0.001 for both survival and metastasis); but the odds ratios for ATA (1.070 [95% confidence interval (CI), 1.030–1.112] for survival and 1.073 [95% CI 1.035–1.112] for metastasis) revealed that they appeared to be useless parameters in practice. However, normalization with RBS made ATA strongly useful. TAR also showed significant associations with survival (*P* = 0.033) and metastasis (*P* = 0.001). TAR showed an odds ratio of 5.931 for survival (95% CI, 1.153–30.513) and 14.144 for metastasis (95% CI, 2.826–70.784). The AUC value for TAR was 0.713 (95% CI, 0.548–0.878) for survival and 0.759 (95% CI, 0.608–0.909) for metastasis, which was superior to that of ATV (0.588 for survival [95% CI, 0.397–0.778] and 0.609 for metastasis [95% CI, 0.431–0.788]) (Figure 3). Apart from associations with 5-years survival and metastasis, TAR also showed significant associations with histological response to chemotherapy, with an odds ratio of 10.746 (95% CI, 1.650–69.989) in Cox regression analysis, while the other parameters did not. In conclusion, TAR on MR images after neoadjuvant chemotherapy was the only parameter that predicted prognosis among parameters for tumor size.

**Table 5:** **Cox regression to evaluate prognostication of tumor sizing parameters**

		5-year survival		Metastasis	
		HR (95% CI)	*P*	HR (95% CI)	*P*
At diagnosis (*n = 41*)	ATL	1.005 (0.995 to 1.015)	0.324	1.005 (0.996 to 1.014)	0.291
	ATV	1.000 (0.997 to 1.003)	0.918	1.000 (0.997 to 1.003)	0.955
	RTV	0.999 (0.994 to 1.005)	0.839	1.000 (0.994 to 1.005)	0.871
	ATA	1.019 (0.988 to 1.050)	0.232	1.024 (0.996 to 1.054)	0.099
	TAR	1.031 (0.153 to 6.950)	0.975	4.748 (0.689 to 32.730)	0.114
After Neoadjuvant Chemotherapy (*n = 57*)	ATL	1.003 (0.994 to 1.012)	0.560	1.004 (0.996 to 1.012)	0.281
	ATV	1.003 (0.997 to 1.009)	0.338	1.004 (0.998 to 1.009)	0.155
	RTV	1.002 (0.992 to 1.012)	0.651	1.005 (0.997 to 1.014)	0.218
	ATA	1.070 (1.030 to 1.112)	<0.001	1.073 (1.035 to 1.112)	<0.001
	TAR	5.931 (1.153 to 30.513)	0.033	14.144 (2.826 to 70.784)	0.001

*Abbreviations*: *HR* hazard ratio, *CI* confidence interval, *ATL* absolute tumor length, *ATV* absolute tumor volume, *RTV* relative tumor volume, *ATA* absolute tumor axial size, *TAR* tumor axial ratio.

In consideration of clinical application, we tried to assign cutoff values to TAR for predicting dichotomous outcomes. The cutoff value was 0.85 for survival and metastasis, which was derived from the Youden index. According to dichotomous predictions validated by two-way contingency table analysis, the accuracy, sensitivity, and specificity of TAR for survival were 75.81% (95% CI, 0.625–0.857), 73.91% (95% CI, 0.559–0.872), and 76.92% (95% CI, 0.663–0.848) respectively, while those for metastasis were 76.36% (95% CI, 0.624–0.865), 72.22% (95% CI, 0.509–0.877) and 78.38% (95% C,. 0.680–0.859).

## Discussion

The size of a primary tumor is regarded as a fundamental parameter for staging solid tumors, but there has been no proven standard method or cutoff value with high performance for predicting prognosis in bone sarcomas. Although many methods and cutoff values have been suggested, most studies have reported on the prognostication of their sizing method using a cutoff value optimized in their study populations. In those studies, there are still questions regarding generalization of the cutoff values. This study is believed to be the first to report a linear correlation (as an odds ratio) between parameter of tumor size and prognosis using successive data analysis in bone sarcomas.

Histological subtypes of osteosarcoma show distinct effects on survival and responses to chemotherapy. In this study, histological responses to chemotherapy were dependent on histological subtype (osteoblastic, chondroblastic, fibroblastic and mixed), validated by *χ*^2^ test (*P* < 0.000). However, there was no significant difference among the four subtypes for survival (*P* = 0.173) and metastasis (*P* = 0.261) in Cox regression analysis. We also evaluated relationships between parameters for tumor size and histological subtype. According to one-way analysis of variance, among the four subtypes, only longitudinal extent, ATL, was significantly different (*P* = 0.020 at diagnosis, *P* = 0.015 after neoadjuvant chemotherapy). Axial extent, TAR (*P* = 0.238 at diagnosis, *P* = 0.527 after neoadjuvant chemotherapy) and 3D ROI volume (*P* = 0.494 at diagnosis, *P* = 0.112 after neoadjuvant chemotherapy) were not.

The significance of the axial extent of a tumor has been considered. The Enneking staging system is also a concern for the axial extent of a tumor [15]. Spanier et al. classified the amount of local axial extension of osteosarcoma as six grades. Multivariate analysis has shown that only the axial extent of a tumor has a significant effect on disease-free survival [16]. Kim et al. reported that longitudinally growing tumors were associated with better survival than concentrically growing tumors in AJCC IIB osteosarcoma [17]. On the basis of these findings, we focused on the axial extent of tumors. However, the application of a method for determining the axial extent of a tumor has limitations with respect to integration into clinical practice. The anatomical relationships between osteosarcoma and surrounding structures differ among affected bones, so the absolute value for invasion axial depth is not applied equally among other affected bones. Although the need for a method to determine relative tumor size has been raised, there is no established universal standard for normalizing tumor size in different bones. BSA is the most widely used method for normalizing tumor size. This approach may be suitable for normalizing tumor burden but not the axial invasion extent of tumors because it does not reflect axial size and spatial relationships between tumors and affected bones. Therefore, we defined new standards for normalizing tumor size from each affected bone by using well-known anatomical landmarks. This adjustment led to significant correlations with prognosis in our study. Absolute parameters for the axial extent of tumors did not show linear correlations with prognosis in this study, but relative parameters did show linear correlations with prognosis.

Many imaging modalities have been suggested to predict response to neoadjuvant chemotherapy and oncological outcomes. The maximum standardized uptake value (SUV_max_) on ^18^ F-fluorodeoxyglucose positron emission tomography (^18^ F-FDG PET) has been reported as an indicator [18-20]. 3D volumetry on MRI with greater precisions compared with previous methods based on the ellipsoid formula has reported reduction of tumor volume as an indicator [4,6]. The apparent diffusion coefficient on diffusion-weighted MRI is also an indicator [21,22]. The major concerns with respect to these indicators are that the values measured after neoadjuvant chemotherapy are more convincing than the values measured at diagnosis. In the present study, TAR showed similar results. TAR measured on MR images at diagnosis was not prognostic, but it was prognostic when measured after neoadjuvant chemotherapy.

Our new method has several advantages over previous methods. First, tumor size is normalized according to the size of the affected bone rather than individual patient size. Normalization with BSA does not discriminate differences in the types of bones involved; however, our method is able to distinguish differences due to bone heterogeneity. Second, our method is easy to use. Calculation of tumor volume using ROI-based analysis on MR images is not practical clinically, but our method requires only measuring the lengths of tumors on the 2D plane of MR images. Third, our method focuses on the extent of tumor axial growth, which results in invasions into the surrounding tissue.

There were several limitations to our study. First, our study was a small retrospective study and only 39 of 59 patients had both MR images at diagnosis and after neoadjuvant chemotherapy, which may have led to wide confidence intervals for the associations of TAR with 5-year survival and metastasis, despite the statistically significant *P* values. This study needs to be validated in larger studies to assess applicability of TAR. Second, the application of our new method was restricted to osteosarcomas located around the epiphysis and metaphysis because RBS was defined by anatomical landmarks around these areas. Thus, our method cannot be applied to diaphyseal and periosteal osteosarcomas. It is also restricted to osteosarcoma located in large joints (shoulder, hip, and knee), although they comprise most cases of osteosarcoma. Third, further optimization of our method by more accurately defining RBS should be considered. Indeed, RBS of the proximal femur was defined as the epiphyseal plate of the femoral head in order to decrease RBS and increase TAR in light of poor prognosis, even though the trochanteric area is more frequently affected than the femoral head.

## Conclusions

TAR is a novel relative sizing method with potential as a prognostic tool in osteosarcoma, which can discriminate differences caused by affected bone heterogeneity as well as individual patient size.

## Abbreviations

AJCC: American Joint Committee on Cancer
ATA: Absolute tumor axial size
ATL: Absolute tumor length
ATV: Absolute tumor volume
AUC: Area under receiver operating characteristic curve
BSA: Body surface area
ICC: Interclass coefficient
RBS: Reference bone axial size
RTV: Relative tumor volume
TAR: Tumor axial ratio
